# Order of draw of blood samples affect potassium results without K-EDTA contamination during routine workflow

**DOI:** 10.11613/BM.2021.020704

**Published:** 2021-04-15

**Authors:** Şerif Ercan, Bahri Ramadan, Ozan Gerenli

**Affiliations:** 1 Department of Medical Biochemistry, Lüleburgaz State Hospital, Kırklareli, Turkey; 2 Department of Anesthesiology and Reanimation, Lüleburgaz State Hospital, Kırklareli, Turkey; 3 Department of Internal Medicine, Lüleburgaz State Hospital, Kırklareli, Turkey

**Keywords:** EDTA contamination, order of draw, patient safety, phlebotomy, preanalytical phase, pseudohyperkalaemia

## Abstract

**Introduction:**

A specific sequence is recommended for filling blood tubes during blood collection to prevent erroneous test results due to carryover of additives. However, requirement of this procedure is still debatable. This study was aimed to investigate the potassium ethylenediaminetetraacetic acid (K-EDTA) contamination in blood samples taken after a tube containing the additive during routine workflow. The study was also carried out to examine the effect of order of draw on potassium results, regardless of K-EDTA contamination.

**Materials and methods:**

In 388 outpatients, to determine the probability of K-EDTA cross-contamination, blood was drawn sequentially into a serum tube, followed by a tube containing K-EDTA, and by another serum tube. In another 405 outpatients, to evaluate the effect of order of draw blood unrelated to K-EDTA contamination, two serum tube were successively collected. Potassium was measured on Cobas 6000 c501 analyser (Roche Diagnostic GmbH, Mannheim, Germany) by indirect ion selective electrode method.

**Results:**

Of paired samples collected before and after a K-EDTA tube, 24% had a potassium difference of above 0.3 mmol/L. However, no EDTA contamination was detected in these samples as well as 95% confidence intervals (CI) of limits of agreement for calcium were within the allowable error limits based on reference change values. Interestingly, of blood samples drawn successively, 24% had also a difference greater than 0.3 mmol/L for potassium.

**Conclusion:**

Incorrect order of draw using closed blood collection system does not cause K-EDTA contamination, even in routine workflow. However, regardless of K-EDTA contamination, order of draw has significant influence on the potassium results.

## Introduction

Spurious hyperkalaemia or pseudohyperkalaemia is a phenomenon frequently encountered in clinical laboratory (*1*). It can occur due to patient-related factors including leukocytosis, thrombocytosis, familial pseudohyperkalaemia, reverse pseudohyperkalaemia (*1*, *2*). There are also several preanalytical variables that affect potassium concentrations artefactually. Haemolysis, traumatic venipuncture, fist clenching, delay in centrifugation are often listed among preanalytical causes of pseudohyperkalaemia (*1*, *2*). Potassium ethylenediaminetetraacetic acid (K-EDTA) contamination is also a significant preanalytical cause of pseudohyperkalaemia (*3*).

Anticoagulant K-EDTA is frequently used as a sample tube anticoagulant for laboratory assays including complete blood count, glycated haemoglobin and blood typing. When blood is collected into a tube with K-EDTA and a serum tube in a single venipuncture, the spurious hyperkalaemia due to K-EDTA contamination has been reported for both open and closed blood collection systems in case reports as well as in studies that aimed to determine frequency of K-EDTA contamination (*4*-*11*). In observational studies, the prevalence of K-EDTA contamination have been variously described as ranging from 3% to 25%. To minimize the probability of K-EDTA contamination in serum samples, in guidelines regarding blood collection practices published by several organizations, it has been recommended that serum tubes should be taken prior to K-EDTA containing tubes (*12*-*14*).

Although findings from epidemiologic studies and case reports shown that K-EDTA contamination is relatively common, it could not been confirmed by the studies that tried to mimic the error regarding order of draw during blood collection (*15*-*18*). In these studies, blood collection has been carried out in ideal phlebotomy conditions from study populations with small sample size, which might be reason of the failure to confirm K-EDTA contamination.

This study, which was designed with large sample size, aimed to investigate the K-EDTA carryover during routine workflow. We also examined the effect of the order of filling the tubes on potassium results without the contamination of K-EDTA by comparing the results obtained from the two serum tubes filled successively.

## Materials and methods

### Subjects

This study was designed as a cross-sectional study. A total of 793 outpatients who have been asked for a blood collection by physician for performing clinical chemistry tests, immunoassay tests and complete blood count in a single venipuncture were enrolled to the study. In our hospital, routinely, blood samples are collected into separate serum tubes for clinical chemistry tests and immunoassay tests. An additional tube of blood was therefore not drawn from any participants in the study. All participants were aged between 18 and 65 years and had no pregnancy status.

The participants were separated into two groups. In the first group (Group 1), which consisted of 388 participants, to determine the probability of K-EDTA cross-contamination during blood collection, blood was drawn sequentially into serum tube with gel separator (8 mL, VACUETTE Z Serum Sep Clot Activator, Greiner Bio-One, Kremsmünster, Austria), followed by a tube containing K-EDTA (2 mL, 3.4 mg K_2_EDTA, BD Vacutainer, Plymouth, UK), followed by another serum tube with gel separator (8 mL, VACUETTE Z Serum Sep Clot Activator, Greiner Bio-One, Kremsmünster, Austria).

In the other group (Group 2) comprised of 405 participants, to evaluate the effect of order of draw blood unrelated to K-EDTA contamination, two serum tubes with gel separator (8 mL, VACUETTE Z Serum Sep Clot Activator, Greiner Bio-One, Kremsmünster, Austria) were filled successively.

All blood samples were drawn from a vein of the upper limb in outpatient collection room during routine workflow using a closed vacuum system consisting of a needle (21 gauge), a tube holder, and the evacuated tubes. The venipuncture was performed by two trained phlebotomist. For patients enrolled to the present study, blood tube which would be used for clinical chemistry tests was labelled with a coloured sticker additional to patient barcode. In this way, phlebotomists had been informed about order of draw which needed to follow. If blood was drawn from the cubital fossa by first attempt, phlebotomists have been requested to sign the sticker.

The research related to human use and complied with all the relevant national regulations, institutional policies and in accordance the tenets of the Helsinki Declaration, and has been approved by Kırklareli Provincial Health Directorate Review Board (Approval Number: 18.07.2019/31).

### Methods

All serum tubes were allowed to clot for 30 minutes at room temperature and then centrifuged at 2000xg for 10 minutes.

Sodium, potassium and chloride were measured on Cobas 6000 c501 analyser (Roche Diagnostic GmbH, Mannheim, Germany) by indirect ion selective electrode method in all serum samples obtained from both groups.

Moreover, in Group 1, if the difference between potassium results obtained from the serum tubes filled before and after the tube with K-EDTA was above 0.3 mmo/L, which is total allowable error limit proposed by Clinical Laboratory Improvement Amendments 2019, serum calcium and EDTA concentrations were also determined in these samples (*19*). Calcium concentrations were measured on Cobas 6000 c501 analyser (Roche Diagnostic GmbH, Mannheim, Germany) by colorimetric assay method using the chromophore 5-nitro-5’-methyl-(1,2-bis(o-aminophenoxy)ethan-N,N,N’,N’-tetraacetic acid (NM-BAPTA). Serum EDTA was assayed on Cobas 6000 c501 analyser (Roche Diagnostic GmbH, Mannheim, Germany) by a previously described method based on its ability to extract copper ions from 1-(2-pyridylazo)-2-naphthol-copper(II) (*20*). For EDTA, the lower limit of detection was 0.05 mmol/L with an analytical range up to 0.5 mmol/L. Lima-Oliveira *et al*. reported that K-EDTA contamination of 0.09 mg/mL (approximately 0.307 mmol/L) have increased potassium concentration by 0.6 mmol/L (*21*). Similarly, Chadwick *et al.* noted that K-EDTA contamination of 0.142 mmol/L could cause a bias of 0.37 mmol/L for potassium (*22*). Therefore, when a K-EDTA contamination increasing potassium concentration of 0.3 mmol/L is present, the method with the lower limit of detection of 0.05 mmol/L for EDTA is able to detect this contamination.

Sodium, potassium, chloride and calcium concentrations were measured using fresh serum samples within 2 hours after blood collection. For EDTA measurements, serum samples were transferred to microtubes and stored at - 20 °C.

For sodium, potassium, chloride and calcium, analytical variation was estimated from analysis of duplicates of the samples that firstly filled during blood collection using the Dahlberg formula (*23*). To determine repeatability and within-laboratory precision for EDTA assay method, the serum samples containing EDTA of 0.15 mmol/L and 0.25 mmol/L were measured during 20 days, with two runs per day and two measurements *per* run (*24*).

For all samples, the haemolysis index (HI) was also quantitatively estimated by bichromatic wavelength paired measurement at 570 and 600 nm on Cobas 6000 c501 analyser (Roche Diagnostic GmbH, Mannheim, Germany). The samples with HI of above 50 (approximately haemoglobin concentration of 0.5 g/L) were excluded from the study.

### Statistical analysis

The statistical significance of the differences between results obtained from serum tubes was determined by the paired samples t-test or the Wilcoxon signed-rank test after identifying whether data was normally distributed or not. Normality was checked by the Shapiro-Wilk test.

For sodium, potassium, chloride and calcium, Bland-Altman plots were also used to describe agreement between the results from the paired serum tubes. If the 95% confidence interval (CI) of upper and lower limits of agreement exceeded the reference change values (RCVs) derived from within-subject biological variation and analytical variation, it was considered clinically significant. Reference change values for increases and decreases in analyte concentration were separately determined using the log normal approach defined by Fokkema *et al.* (*25*). Biological variation values were obtained from The European Federation of Clinical Chemistry and Laboratory Medicine European Biological Variation Study (*26*). Within-subject biological variation values have been reported to be 0.53%, 3.92%, 0.98% and 1.81% for sodium, potassium, chloride and calcium, respectively (*26*).

All statistical analyses were performed using MedCalc Statistical Software version 19.1 (MedCalc Software Ltd, Ostend, Belgium) and Minitab 17 (Minitab, LLC, Pennsylvania, USA). All P values less than 0.05 were considered to be statistically significant.

## Results

In Group 1, the median age of participants was 48 and 77% was female. In Group 2, similarly, the median age of participants was 47 and 78% was female.

Analytical variation was determined 0.7%, 0.62%, 0.53% and 1.45% for sodium, potassium, chloride and calcium, respectively, using the results obtained from duplicate analysis of the serum tubes that filled before the tube with K-EDTA. The repeatability of EDTA assay method was estimated to be 4.01% and 3.14% in the sample containing EDTA of 0.15 mmol/L and 0.25 mmol/L, respectively. The within-laboratory precision for EDTA was also determined to be 5.04% and 4.81% for samples with EDTA of 0.15 mmol/L and 0.25 mmol/L, respectively.

Reference change values for decreases/increases in analyte concentration were estimated as - 2.0% / 2.1%, - 8.8% / 9.7%, - 2.6% / 2.6% and - 5.3% / 5.6% for sodium, potassium, chloride and calcium, respectively.

In Group 1, 5 participants were excluded since the HI values were above than 50 in one of paired serum samples. In this way, the effect of haemolysis on potassium results was eliminated for all serum samples. The sodium, potassium and chloride results from a total of 383 subjects were used in final data analysis.

**Figure 1:**
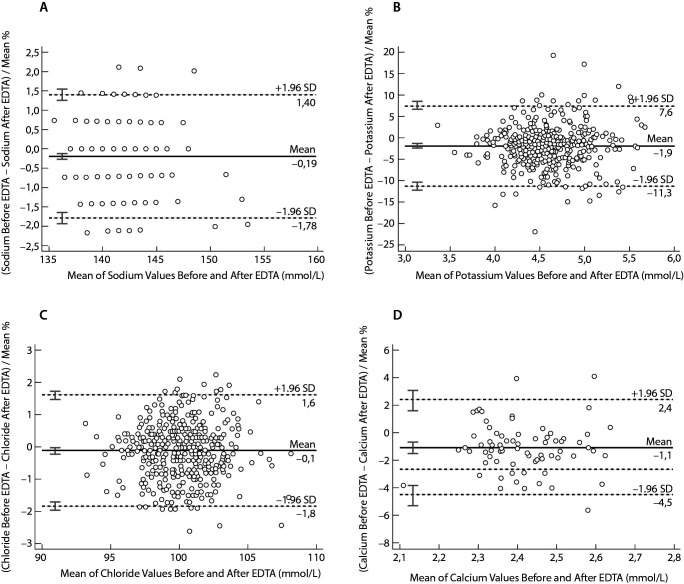
The Bland-Altman plots comparing the results of sodium (A), potassium (B), chloride (C) and calcium (D) obtained from the serum tubes filled before and after the tube with K-EDTA. The straight line indicates the mean difference with 95% confidence intervals, the deshed lines shows the upper and lower limits of agreement with 95% confidence intervals. K-EDTA - potassium ethylenediaminetetraacetic acid.

Table 1 shows the mean bias between the results obtained from the serum tubes filled before and after the tube with K-EDTA. In paired statistical analysis, there was a significant difference for sodium (P < 0.001), potassium (P < 0.001), and chloride (P = 0.021). In Bland-Altman analysis, 95% CI of limits of agreement were found to be lower for sodium and chloride than allowable error limits based on RCVs. However, for potassium, the lower limit of agreement was determined to be - 11.3%, which is higher than acceptable limit based on RCV. Bland-Altman plots are shown in Figure 1.

**Table 1:** Results of investigated parameters in blood samples collected before and after the tube containing K-EDTA

**Analyte**	**Before EDTA,** **Mean ± SD**	**After EDTA,** **Mean ± SD**	Mean Difference (CI)*
Sodium (mmol/L)	141.5 ± 2.4	141.8 ± 2.5	- 0.27 (- 0.39 to - 0.16)^†^
Potassium (mmol/L)	4.56 ± 0.39	4.65 ± 0.39	- 0.09 (- 0.11 to - 0.06)^†^
Chloride (mmol/L)	100.1 ± 2.4	100.2 ± 2.4	- 0.11 (- 0.20 to - 0.02)^‡^
Calcium (mmol/L)	2.4 ± 0.1	2.43 ± 0.1	- 0.03 (- 0.04 to - 0.01)^†^
EDTA (mmol/L)	< 0.05	< 0.05	/
Haemolysis Index	14.9 ± 8.4	13 ± 6.9	/
*The difference of test results obtained from the paired samples was statistically evaluated by paired t-test (for calcium) or Wilcoxon signed ranks test (for sodium, potassium and chloride) (^†^P < 0.001, ^‡^P < 0.05). SD - standard deviation, CI - confidence interval. EDTA - ethylenediaminetetraacetic acid.			

In addition, EDTA and calcium concentrations were measured in 92 paired serum tubes that was observed a difference of above 0.3 mmol/L for potassium. Although there was a significant difference for calcium in paired statistical analysis (P < 0.001), 95% CI of limits of agreement were found to be within allowable error limits (Table 1) (Figure 1). Of these 92 paired samples, none had detectable EDTA concentration which was 0.05 mmol/L.

In Group 2, 6 participants were excluded since the HI values were above than 50 in one of paired serum samples. Therefore, the sodium, potassium and chloride results from a total of 399 subjects were included in data analysis.

The mean differences between the paired serum tubes are shown in Table 2. By paired statistical analysis, when compared the results of sodium, potassium and chloride obtained from the serum tubes consecutively filled, a significant difference have been found for potassium (P < 0.001) and chloride (P = 0.014), but not sodium (P = 0.096).

**Table 2:** Serum sodium, potassium and chloride values in blood samples drawn successively

**Analyte**	**First tube,** **Mean ± SD**	**Second tube, Mean ± SD**	Mean Difference (CI)*
Sodium (mmol/L)	140.9 ± 2.4	140.7 ± 2.4	0.1 (- 0.01 to 0.22)
Potassium (mmol/L)	4.57 ± 0.34	4.7 ± 0.36	- 0.12 (- 0.14 to - 0.1)^†^
Chloride (mmol/L)	99.7 ± 2.4	99.6 ± 2.4	0.1 (0 to 0.2)^‡^
Haemolysis Index	15.9 ± 7.7	16 ± 7.9	/
*The difference of test results obtained from the paired samples was statistically evaluated by paired t-test (for calcium) or Wilcoxon signed ranks test (for sodium, potassium and chloride) (^†^P < 0.001, ^‡^P < 0.05). SD - standard deviation, CI - confidence interval.			

**Figure 2:**
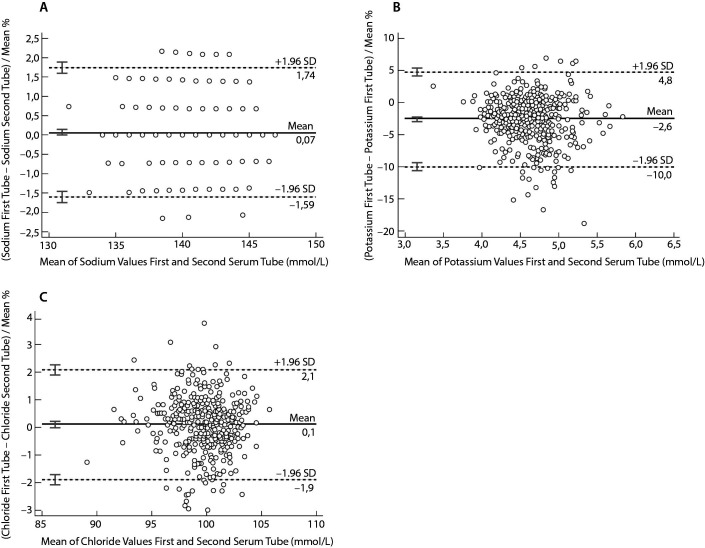
The Bland-Altman plots for sodium (A), potassium (B) and chloride (C) from blood samples collected successively. The straight line indicates the mean difference with 95% confidence intervals, the dashed lines show the upper and lower limits of agreement with 95% confidence intervals.

In Figure 2, Bland-Altman plots shows that 95% CI of limits of agreement were within the allowable error limits based on RCV for sodium and chloride. However, for potassium, the lower limit of agreement (10%) was found to be beyond acceptable limit.

## Discussion

In the present study, clinically significant difference was found for potassium results obtained from the serum tubes filled before and after the tube containing K-EDTA. A total of 18% of blood samples collected after the tube containing K-EDTA had higher potassium values of at least 0.3 mmol/L than those of the samples collected before. Moreover, higher potassium values were encountered in 6% of the serum tubes taken before K-EDTA by at least 0.3 mmol/L. However, any trace of EDTA concentration was not determined in any of these samples. In addition, there was no clinically significant difference between calcium results from these samples. Interestingly, similar to the findings from comparing the results obtained from the serum tubes filled before and after the tube containing K-EDTA, it was also determined a difference higher than 0.3 mmol/L for potassium in 24% of blood samples drawn successively.

A few previous studies, in controlled laboratory environment, aimed to investigate whether the incorrect order of draw result in EDTA contamination by comparing the results from the tubes filled before and after the tube with EDTA. Majid *et al.* compared potassium and calcium results from the vacuum tubes (Becton Dickinson) containing no anticoagulant filled before and after the tube containing EDTA in 34 inpatients (*15*). They also assessed the difference between serum tubes filled sequentially. In their study, it has been found that there was no significant difference between non-anticoagulated sample pairs for both of two groups.

In another study comprised of 10 healthy volunteers, Sulaiman *et al.* reported that the blood collection for clinical chemistry analytes after an EDTA sample using Sarstedt S-Monovette venipuncture system has no effect on potassium and calcium results as well as other analytes measured (*16*). They also measured EDTA concentrations in all serum samples and could not detect any trace of it.

Similar to these findings, Cornes *et al*. noticed that potassium and calcium values were undistinguished in blood samples drawn before and after the tube with K-EDTA from 11 healthy volunteers using the Becton Dickinson Vacutainer system (*17*). They have also determined an EDTA concentrations of below 0.2 mmol/L in all samples.

In a more recent study which consisted of 58 healthy volunteers, the findings supporting those of previous studies in this field have been reported by Salvagno *et al*. using Terumo Europe Venosafe evacuated blood tubes (*18*). They suggested that the order of draw has a negligible effect on sample quality and should not be used as a quality criterion when evaluating the performance of phlebotomist.

Our findings regarding EDTA and calcium is in concordance with those of the previous studies (*15*-*18*). However, for potassium, our results differ from those of these studies. For potassium, the difference in the findings from the present study with those of previous studies may be related to sample size of studies. Our study have a large sample size with 383 subjects, whereas sample size of others was ranging from 10 to 58. Conditions contributing to the difference in potassium results may differ among individuals. To determine the cumulative effect of these, larger sample size is more appropriate. On other hand, in previous studies, blood collection have been performed from healthy volunteers in ideal phlebotomy conditions (*16*-*18*). In contrast, the current study consisted of outpatients with blood collection conducted in routine workflow.

Majid *et al.* suggested that the effect of difficult venipuncture, which lead to local tissue damage and then induces release of potassium from damaged cells, is more important than order of draw of blood samples (*15*). This may partly explain the bias determined for potassium in the present study. We thought that local tissue damage might occur, even if difficult venipuncture is not a case, and might reach a degree altering potassium results. There are inter-individual variability in diameter of superficial veins of the upper arm as well as in the thickness of the surrounding tissues, which could cause inter-individual variation in local tissue damage (*27*).

Another factor contributing to occurrence of difference in potassium results in the present study might be variability in tourniquet application duration and strength during routine workflow. Lippi *et al.* reported that venous stasis by prolonged application of a tourniquet during blood collection could cause decreased potassium results (*28*). In contrast, in another study, elevated potassium results was reported related to the tourniquet induced venous stasis (*29*). The guidelines regarding blood collection recommends that the release of the tourniquet should be carried out as soon as the blood flows into the first tube (*13*, *14*). However, this may not be possible at all times if multiple specimens are obtained from a patient. Moreover, in situations at which the tourniquet is not released after blood begins to flow, the mixing of the tube containing K-EDTA, which is necessary to prevent the clotting of collected blood, could lead to filling of first and second serum tube at different tourniquet times. Unfortunately, we have no data regarding the release moment of tourniquet during blood collection in the present study.

In the current study, the nurses asked the patients to forms a fist, but not pumping, during phlebotomy to make the veins more prominent. Although this practice is common and is not in contradiction with recommendations of guidelines regarding blood collection procedures, to standardize the strength of fist as well as duration is not easy during phlebotomy (*12*-*14*). In a study conducted by Seimiya *et al.*, when blood collection was performed after fist making, in 25.6% of samples, potassium concentrations have been found to be higher in the first serum tube than those of third tube by at least 0.2 mmol/L (*30*). Therefore, making fist during phlebotomy might be a reason for elevated potassium concentrations in the first serum tube in the present study.

For sodium and chloride, no clinically significant differences were found between the results from the serum tubes collected before and after the tube with K-EDTA as well as collected successively. There are limited studies comparing sodium and chloride results obtained from the serum tubes drawn in different order. Our findings were in line with those of earlier studies for sodium and chloride (*18*, *30*).

This study have some limitations. Using only one brand of evacuated serum tubes is one of limitations. In the current study, blood collection was performed using Greiner Bio-One blood collection systems, which has not been investigated in previous studies (*15*-*18*). It seems that incorrect order of draw does not cause carryover of K-EDTA regardless of brand of evacuated blood tubes used. However, we have no data regarding whether the findings of this study may be applicable to evacuated blood tubes with different brand or not. Another limitation is that blood drawing was only carried out using the evacuated blood collection system consisted of tube holder and needle. Therefore, the findings of this study may not be applicable to other blood collection systems including syringe or winged blood collection device.

In conclusion, the reversed order of draw using closed blood collection system does not cause carryover of K-EDTA, even in routine workflow. However, irrespective of K-EDTA contamination, order of draw has significant influence on the potassium results. Following the order of draw during blood collection have potential to diminish this effect.
